# Assessing environmental gradients in relation to dark CO_2_ fixation in estuarine wetland microbiomes

**DOI:** 10.1128/aem.02177-24

**Published:** 2024-12-31

**Authors:** Luise Grüterich, Jason Nicholas Woodhouse, Peter Mueller, Amos Tiemann, Hans-Joachim Ruscheweyh, Shinichi Sunagawa, Hans-Peter Grossart, Wolfgang R. Streit

**Affiliations:** 1 Department of Microbiology and Biotechnology, University of Hamburg 14915 , Hamburg, Germany; 2 Department of Plankton and Microbial Ecology, Leibniz Institute of Freshwater Ecology and Inland Fisheries 28401 , Stechlin, Germany; 3 Institute of Landscape Ecology, University of Münster 27193 , Münster, Germany; 4 Department of Biology, Institute of Microbiology and Swiss Institute of Bioinformatics, ETH Zurich 30845 , Zurich, Switzerland; 5 Institute of Biochemistry and Biology, Potsdam University 163276 , Potsdam, Germany; Georgia Institute of Technology, Atlanta, Georgia, USA

**Keywords:** Wood-Ljungdahl pathway, reverse TCA cycle, metagenomics, metatranscriptomics, Elbe estuary, C-cycle, carbon dioxide fixation

## Abstract

The rising atmospheric concentration of CO_2_ is a major concern to society due to its global warming potential. In soils, CO_2_-fixing microorganisms are preventing some of the CO_2_ from entering the atmosphere. Yet, the controls of dark CO_2_ fixation are rarely studied *in situ*. Here, we examined the gene and transcript abundance of key genes involved in microbial CO_2_ fixation along major environmental gradients within estuarine wetlands. A combined multi-omics approach incorporating metabarcoding, deep metagenomic, and metatranscriptomic analyses confirmed that wetland microbiota harbor four out of seven known CO_2_ fixation pathways, namely, the Calvin cycle, reverse tricarboxylic acid cycle, Wood–Ljungdahl pathway, and reverse glycine pathway. These pathways are transcribed at high frequencies along several environmental gradients, albeit at different levels depending on the environmental niche. Notably, the transcription of the key genes for the reverse tricarboxylic acid cycle was associated with high nitrate concentration, while the transcription of key genes for the Wood–Ljungdahl pathway was favored by reducing, O_2_-poor conditions. The transcript abundance of the Calvin cycle was favored by niches high in organic matter. Taxonomic assignment of transcripts implied that dark CO_2_ fixation was mainly linked to a few bacterial phyla, namely, *Desulfobacterota*, *Methylomirabilota*, *Nitrospirota*, *Chloroflexota*, and *Pseudomonadota*.

**IMPORTANCE:**

The increasing concentration of atmospheric CO_2_ has been identified as the primary driver of climate change and poses a major threat to human society. This work explores the mostly overlooked potential of light-independent CO_2_ fixation by soil microbes (a.k.a. dark CO_2_ fixation) in climate change mitigation efforts. Applying a combination of molecular microbial tools, our research provides new insights into the ecological niches where CO_2_-fixing pathways are most active. By identifying how environmental factors, like oxygen, salinity and organic matter availability, influence these pathways in an estuarine wetland environment, potential strategies for enhancing natural carbon sinks can be developed. The importance of our research is in advancing the understanding of microbial CO_2_ fixation and its potential role in the global climate system.

## INTRODUCTION

Rising atmospheric concentrations of the greenhouse gases (GHGs) CO_2_ and methane are primary drivers of climate change and hence a major concern to society. Therefore, knowledge of fundamental processes linked to CO_2_-producing and CO_2_-fixing metabolisms is crucial (1, 2). Within this framework, current research focuses on GHG-fixing microbial metabolic pathways that could be relevant to the climate system because of their ability to counteract the increasing atmospheric GHG concentrations.

Light-driven photosynthesis and the associated CO_2_ fixation pathways (i.e., Calvin cycle) are well understood and play an obvious role in the climate system (3, 4). Although the Calvin cycle contributes to light-dependent photosynthesis, it should be noted that it can operate independently of light in autotrophic microorganisms, such as sulfur-oxidizing bacteria and methylotrophs (5, 6). In addition to light-dependent CO_2_ fixation, also non-photosynthetic GHG fixation pathways can play a major role in the climate system. One prominent example of non-photosynthetic microbial GHG fixation is methanotrophy. Aerobic and anaerobic methane oxidizers are responsible for preventing up to 90% of methane produced in soils or aquatic systems from entering the atmosphere; the number varies depending on the ecosystem (7–9). Additionally, the conversion of methane to CO_2_ by methanotrophs underscores the complexity of microbial interactions influencing soil–atmosphere GHG fluxes. Extensive research has refined our quantitative and mechanistic understanding of methanotrophy and its role in the global climate system (8, 10, 11). By contrast, while pure culture studies have been conducted extensively, research on *in situ* studies of dark CO_2_ fixation is limited. Most of these studies have focused on single-species pure cultures (e.g., [12, 13]), with only a few investigations exploring this process in natural environments, such as paddy soils, semiarid deserts, and grasslands (14–17). Thus, the environmental controls of dark CO_2_ fixation and its potential effects on the global climate system remain largely unknown. Recently, however, this topic receives increasing attention from the scientific community (12, 18). Among the domains of life, at least seven autotrophic CO_2_ fixation pathways can be distinguished: Calvin cycle, reductive tricarboxylic acid cycle (rTCA cycle), Wood–Ljungdahl pathway (WLP), 3-hydroxypropionate bicycle (3-HP bicycle), 3-hydroxypropionate/4-hydroxybutyrate cycle (3-HP/4-HB cycle), dicarboxylate/4-hydroxybutyrate cycle (DC/4-HB cycle), and reductive glycine pathway (rGlyP) (19–22) (Table 1). Importantly, a recent study reported that high levels of CO_2_ drive the TCA cycle backward toward autotrophy (18). Due to the increase in CO_2_ concentration in the atmosphere mainly caused by anthropogenic fossil fuel burning, it is now crucial to elucidate the environmental factors that determine the occurrence and magnitude of the respective dark CO_2_ fixation pathways *in situ*.

**TABLE 1:** List of CO_2_-fixing pathways and affiliated key genes^*a*^

Pathway	Key enzyme	Key gene also present in another CO_2_-fixing pathway^*b*^	Key gene for abundance analysis	EC number
rTCA cycle	2-Oxoglutarate synthase	-		1.2.7.3
	ATP-citrate lyase*	-	*aclA*	2.3.3.8
WLP	Acetyl-CoA synthase*	-	*acsC/cdhE*	2.3.1.169/2.1.1.245
	CO dehydrogenase	-		1.2.7.4
Calvin cycle	RuBisCO*	-	*rbcL*	4.1.1.39
	Phosphoribulokinase	-		2.7.1.19
DC/4-HB cycle	4-Hydroxybutyryl-CoA dehydratase	3-HP/4-HB cycle		4.2.1.120
	Malate dehydrogenase	rTCA cycle		1.1.1.37
3-HP bicycle	Malonyl-CoA reductase	3-HP/4-HB cycle		1.2.1.75
	Propionyl-CoA synthase	3-HP/4-HB cycle		6.4.1.3
	Malyl-CoA lyase	-		4.1.3.24
3-HP/4-HB cycle	Acetyl-CoA/propionyl-CoA carboxylase	3-HP bicycle		6.4.1.3
	Methylmalonyl-CoA mutase	3-HP bicycle		5.4.99.2
	4-Hydroxybutyryl-CoA dehydratase	DC/4-HB cycle		4.2.1.120
rGlyP	Glycine reductase*	-	*grdB*	1.21.4.2
	Methenyl-THF cyclohydrolase	-		3.5.4.9

^*a*^ The selection of key genes is based on Berg *et al.* (21) and was supplemented by the malate dehydrogenase for the DC/4-HB cycle and the key genes for the rGly pathway (22). Asterisks indicate the key genes selected for analyzing gene and transcript abundance (Fig. 4) as well as for the correlation analysis of transcript abundance with environmental parameters (Table 3).

^*b*^ Hyphen means "does not apply" .

**Fig 1:**
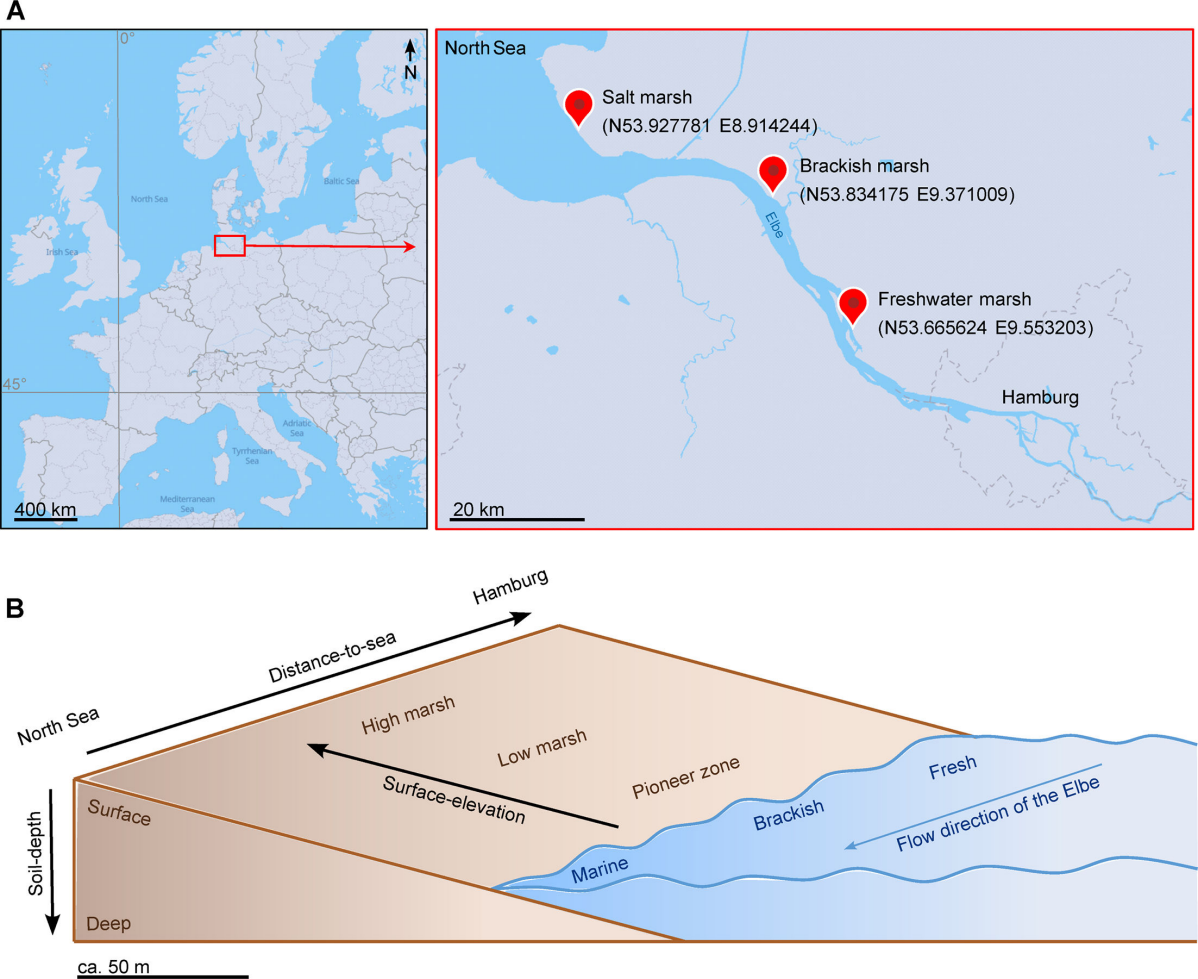
Location of the Elbe estuary and sampling sites along the distance-to-sea gradient of the Elbe estuary from the salt marsh to the brackish marsh to the freshwater marsh (**A**). Environmental gradients given by the sampling locations along the river course from fresh to brackish to marine sites (distance-to-sea gradient), along the surface-elevation gradient from pioneer zones to low marshes to high marshes, and the soil-depth gradient we sampled (surface layer [0–5 cm] versus deep layer [45–50 cm]) (B). At each site, in each zone and depth, samples were taken with a peat sampler in September at low tide. Maps in A were created with MapTiler.

The present study assessed the gene and transcript abundance of key genes of the different dark CO_2_-fixing pathways along environmental gradients within an estuarine wetland landscape to provide insight into which CO_2_-fixing pathways are most likely to be present in different environmental niches. With this, we aim to create a basis on which further in-depth research on dark CO_2_ fixation in the environment can be built. The estuarine wetland landscape is ideally suited to understand environmental constraints on CO_2_-fixing pathways for at least two reasons. First, estuarine wetlands connect marine, limnic, and terrestrial environments, and are therefore characterized by steep environmental gradients in oxygen availability, organic matter availability, and salinity. Second, estuarine wetland soils possess a tremendous carbon turnover rate and are among the most effective carbon sinks of the biosphere (23, 24). Our study was conducted along the salinity gradient of the Elbe estuary, NW Germany. The Elbe estuary with a coverage of 500 km^2^ is one of the largest estuaries in Europe with various environmental gradients. For this study, we sampled along multiple small- and large-scale spatial gradients within the estuary, distance to sea, surface elevation, and soil depth, encompassing three sampling sites ranging from marine to freshwater marshes (Fig. 1).

We assessed the following two hypotheses: (I) Dark CO_2_ fixation occurs in anaerobic ecological niches with low reduction potential because many of the CO_2_-fixing pathways have been described in anaerobic microorganisms (19). (II) Dark CO_2_ fixation benefits from heterotrophic respiratory CO_2_ production, which we expect to increase with increasing salinity in anaerobic environments, associated with the greater supply of alternative electron acceptors, i.e., sulfate, facilitating anaerobic heterotrophic respiration (25, 26).

While some studies have examined the gene and transcript abundance of key genes for CO_2_ fixation pathways in specific ecosystems or under specific environmental conditions, this study investigates transcript abundance across large- and small-scale environmental gradients, allowing for the identification of niches of these pathways.

## RESULTS

In order to constrain the ecological niches of dark CO_2_-fixing pathways *in situ*, the soil microbiome was assessed along different gradients within the marshes of the Elbe estuary: distance to sea, surface elevation, and soil depth (Fig. 1).

We measured several environmental parameters along these spatial gradients, namely, organic matter content, reduction index (as proxy for O_2_ availability), chloride (as proxy for salinity), nitrate, sulfate, acetate, pyruvate, and isocitrate. We expected the availability of sulfate, as the dominant alternative electron acceptors for anaerobic heterotrophic respiration in marine and estuarine environments, to covary with the distance-to-sea gradient. We were able to confirm that, indeed, sulfate and chloride decreased significantly (*P* < 0.05) with increasing distance to sea. We further observed that oxygen availability and organic matter content decreased significantly (*P* < 0.05) with increasing soil depth and that concentration of isocitrate increased significantly (*P* < 0.05) with higher distance to sea (Table 2; Table S3).

**TABLE 2:** Statistical analysis of environmental parameters correlating with the spatial gradients distance to sea, surface elevation, and soil depth (Fig. 1)^*a*^

	Chloride	Organic Matter	Acetate	Pyruvate	Isocitrate	Nitrate	Sulfate	Reduction
Distance to sea	**−0.77**	0.24	0.42	0.20	**0.61**	0.16	**−0.69**	0.22
Surface elevation	−0.13	−0.31	−0.01	−0.28	0.05	0.27	−0.18	−0.11
Soil depth	0.12	**−0.75**	−0.26	0.46	0.12	−0.24	0.08	**0.78**

^*a*^ Significant changes are indicated by bold numbers (*P* < 0.05). We performed a Spearman’s rank correlation analysis with transcript abundances of the key genes and eight soil environmental parameters. A positive number indicates increase and a negative number indicates decrease in the measured environmental parameters. Given numbers represent Spearman's *ρ* (metadata in Table S3).

**Fig 2:**
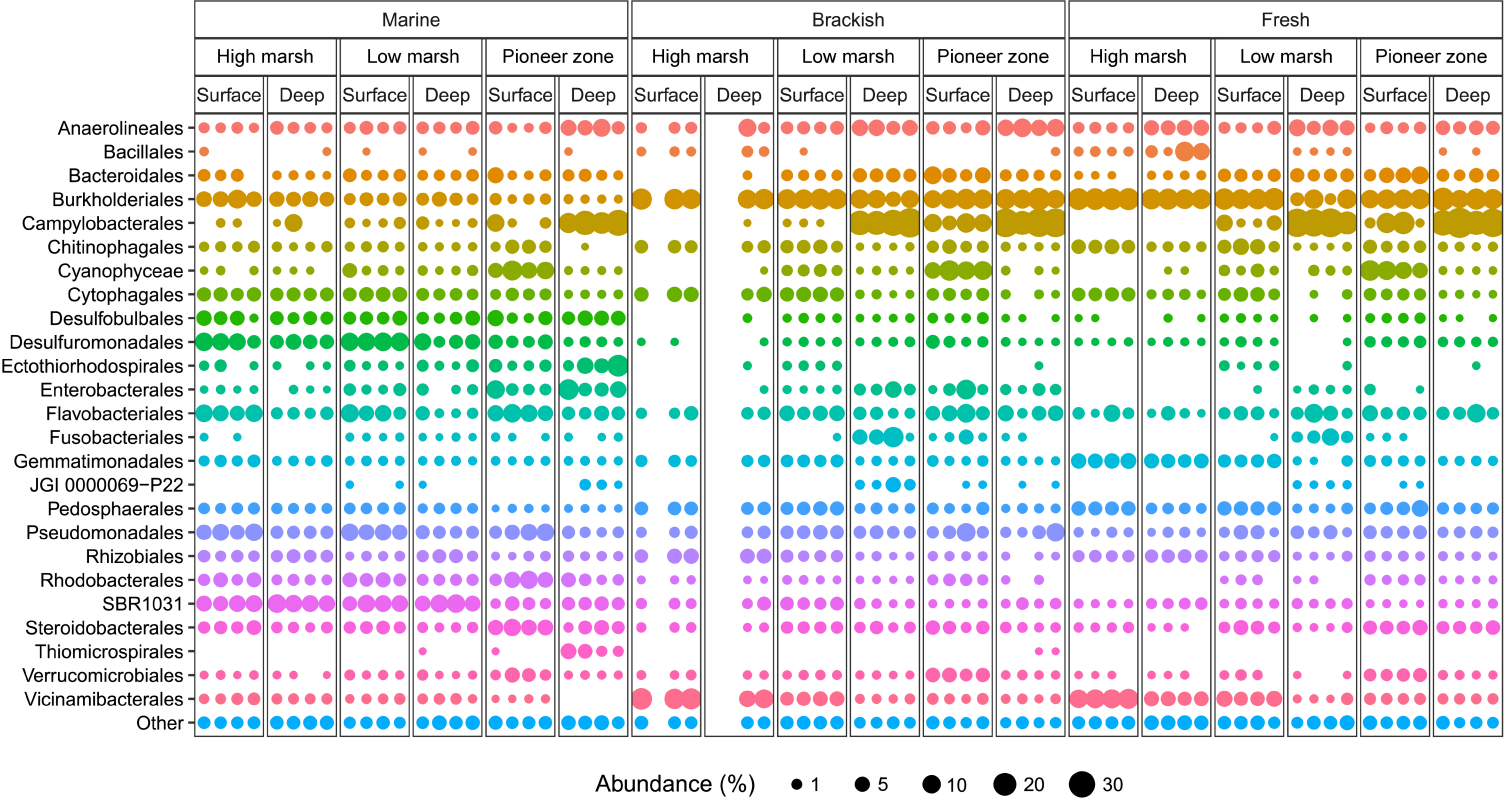
Taxonomic composition of bacterial communities at the order level for the 18 sampling locations with four biological replicates (four different cores with 2 m distance each), resulting in a total of 69 analyzed samples (three replicates could not be included due to insufficient sequence quality). Samples originate from the three sites: Salt marsh (marine), brackish marsh (brackish), and freshwater marsh (fresh). Each site was sampled at the three zones: High marsh, low marsh, and pioneer zone. Each zone was sampled at the surface layer at 0 cm (surface) and the deep layer at 50 cm depth (deep). Depicted are the 25 orders with the highest average relative abundances. All other orders were combined into the “other” group.

We assessed the extent to which wetland microbiota harbor the potential for dark CO_2_ fixation, and how gene and transcript abundance vary along environmental gradients in distance to sea, surface elevation, and soil depth (Fig. 1). In total, our sampling design includes 69 amplicon data sets (Fig. 2) as well as 18 metagenome and the corresponding metatranscriptome data sets (Fig. 4) with associated organic matter, reduction index, chloride, nitrate, sulfate, acetate, pyruvate, and isocitrate data (Table 2; Table S3). All underlying samples were collected in September. Thus, all resulting data represent a snapshot at this time of year.

### Community composition shifts of wetland microbiota

Phylogenetic analysis was performed to obtain further insight into the metabolic capabilities of wetland microbiota (27, 28). Samples were taken as soil cores at 18 locations, and DNA and RNA were extracted as specified in the Material and Methods. The 16S rRNA amplicon sequencing results of all 69 samples indicated that the top four most abundant orders were *Campylobacterales* with an average of 10.2%, *Burkholderiales* with an average of 9.7%, *Flavobacteriales* with an average of 3.9%, and *Anaerolineales* with an average of 3.3% (Fig. 2). *Campylobacterales*, *Burkholderiales*, and *Anaerolineales* harbor species capable of CO_2_ fixation (29–31). Comparing the abundance of the 25 most abundant orders, shifts in community composition occur along the different environmental gradients (Fig. 2).

16S rRNA provides the advantage of observing multiple functional group shifts simultaneously. However, connecting phylogenetic groups to function is challenging due to microbial plasticity and functional redundancy. Limited knowledge exists about the impact of microbial composition on community-level physiological profiles (32).

### Metagenomics show high potential for microbial CO_2_ fixation in estuarine wetlands

**Fig 3:**
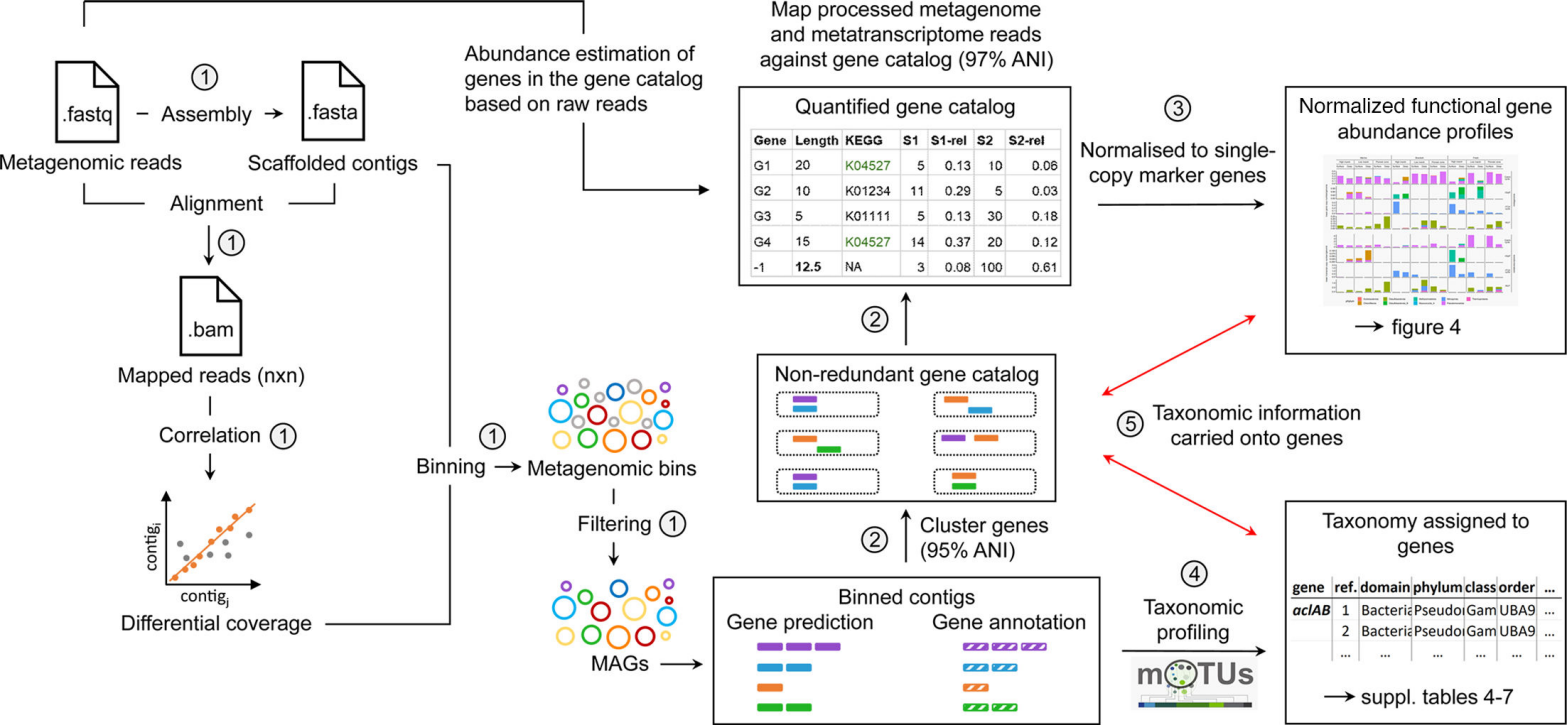
Schematic overview of sequence analyses using a gene catalog pipeline for metagenomic and metatranscriptomic reads. (1) Processed reads from each sample were assembled independently, and all samples were used to guide per sample binning. (2) Gene prediction and annotation were performed on all prokaryotic metagenome-assembled genomes (MAGs), and a non-redundant gene catalog was built using a clustering threshold of 95% ANI. (3) Processed metagenome and metatranscriptome reads were mapped against the gene catalog and normalized based on gene length (within sample normalization) and single-copy marker gene abundance (between sample normalization). (4) MAGs were placed into species level mOTUs using single-copy marker genes, and assigned taxonomy using GTDB-TK, used for accurate taxonomic profiling. (5) Information linking taxonomy from MAGs and gene/transcript abundance was maintained at all times to allow for taxonomic interrogation of functional gene abundance.

The analyses presented above reveal a high phylogenetic diversity, which strongly suggests a corresponding high metabolic diversity within the marsh soil microbiota. While many of the identified orders harbor species capable of CO_2_ fixation (29–31), we wanted to assess the functional genetic potential for dark CO_2_ fixation along environmental gradients (Fig. 1B). We sequenced 18 metagenomes. For each sample, a minimum of 54 million and an average of 99 million reads were obtained with an average N50 value of 1,173 (Table S1). A schematic overview of the bioinformatic workflow is provided below (Fig. 3).

**Fig 4:**
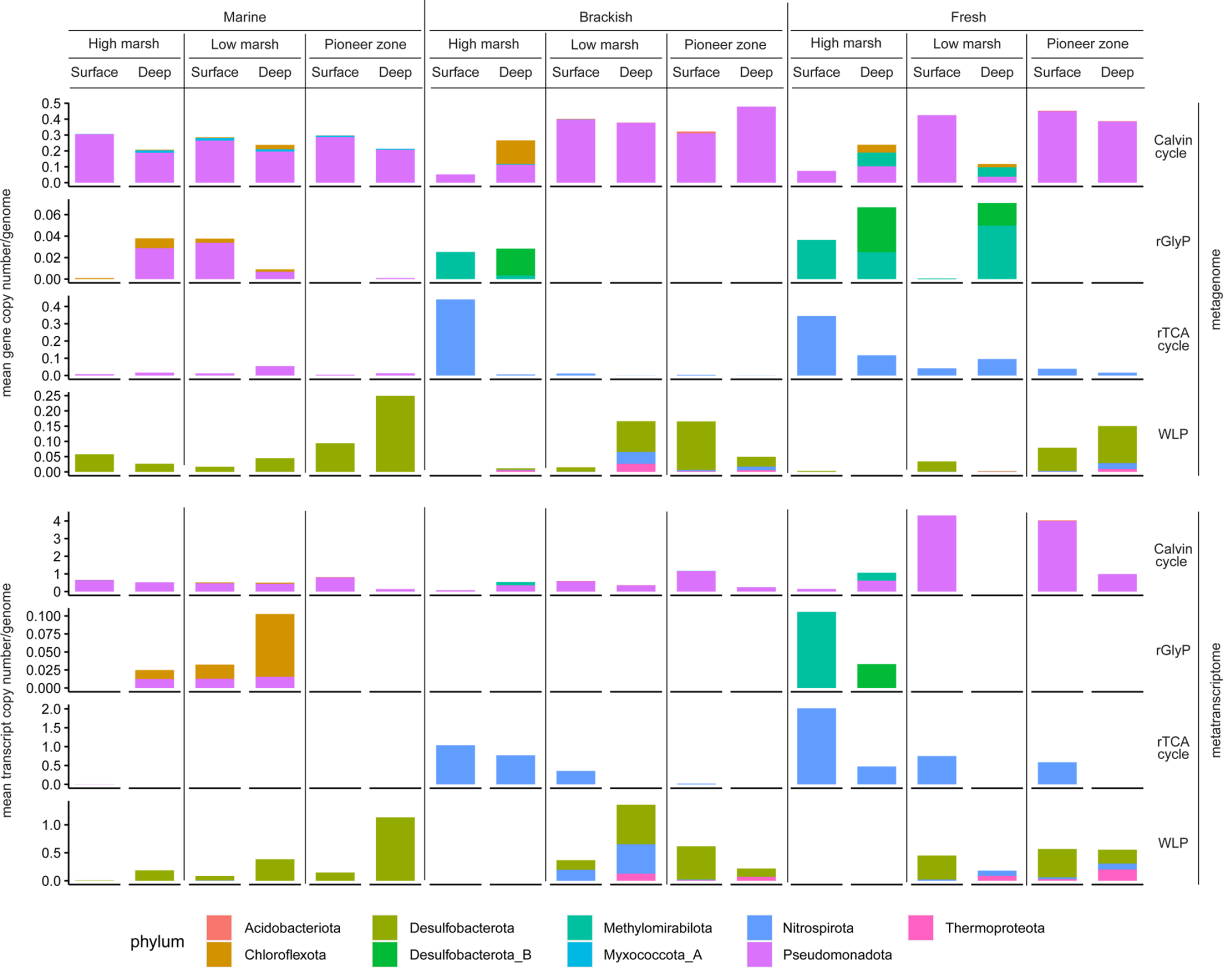
Metagenome and metatranscriptome analysis of CO_2_-fixing pathways. Abundance of the key genes (copy number per genome) (metaG) ATP-citrate lyase (for rTCA), acetyl-CoA synthase (for WLP), RuBisCO (for Calvin cycle), glycine reductase (for rGlyP), and their respective transcripts (metaT) normalized to single-copy marker gene. Colors represent the respective microbial groups of the MAGs to which the key genes were assigned to. The 18 depicted samples originate from the three sites: Salt marsh (Marine), brackish marsh (Brackish), and freshwater marsh (Fresh) reflecting the distance-to-sea gradient. Each from the three zones: High marsh, low marsh, and pioneer zone reflecting the surface-elevation gradient. Each from the surface layer at 0–5 cm (Surface) and the deep layer at 45–50 cm depth (Deep) reflecting the soil-depth gradient.

On the basis of the obtained metagenome data, we analyzed the relative abundance of key genes for the metabolic pathways involved in microbial CO_2_ fixation. We aimed to develop a pipeline using multiple genes, genomic context, and taxonomic information to assess the completeness of dark CO_2_ fixation pathways. Detailed information on this pipeline is provided in the Material and Methods section and the supplement (Text S1; Fig. 4; Table 1).

Based on this approach, we were able to detect the presence of four out of seven CO_2_-fixing pathways, although with varying copy numbers along the environmental gradients (Fig. 1B). The copy number of the four key genes that belong to the four present CO_2_-fixing pathways in our samples, ranged from a mean gene copy number/genome of 0 to 0.48.

The rGlyP had the lowest abundance of the four present CO_2_-fixing pathways with a mean gene copy number of 0 to 0.07 per genome. The most frequently detected pathway was the Calvin cycle, which had copy numbers ranging from 0.05 to 0.48. (Fig. 4).

The Calvin cycle was mainly assigned to MAGs belonging to the phylum *Pseudomonadota*. The taxonomy behind the rGlyP appeared to be more diverse and depending on the environmental niche. *Chloroflexota* and *Methylomirabilota* were the most prominent representatives of the rGlyP. The rTCA cycle was mainly assigned to the phylum of *Nitrospirota*. The WLP was predominantly assigned to *Desulfobacterota*.

Considering the number of shared genes/enzymes between the 3-HP bicycle, the 3-HP/4-HB cycle, and the DC/4-HB cycle, their overlap with other carbon-fixing pathways and the potential redundancy of genes with other metabolic pathways, ultimately proving the functionality of these pathways from metagenome data is tenuous. Despite this, we still sought to identify genomes where these pathways are present and use these data to explore their ecological significance. Based on the complete absence of the relevant genes and transcripts from any genome, we considered that the 3-HP bicycle, 3-HP/4-HB cycle, and the DC/4-HB cycles were completely absent from genomes in our samples.

### Metatranscriptomics reveal high transcript abundance of key genes related to microbial CO_2_ fixation

Based on the observation that Calvin cycle, rGlyP, rTCA, and WLP were present along the investigated environmental gradients (Fig. 1B and 4), we asked to what extent these genes were transcribed. Therefore, we analyzed the transcription level of the key genes involved in these pathways (Table 1; Fig. 4). RNA from a total of 18 soil samples was extracted as described in the Material and Methods section, used for mRNA sequencing, and mapped to the metagenome (Table 1; Fig. 4). For each sample, a minimum of 50 million and an average of 80 million reads were obtained with an average N50 value of 750 (Table S1). We detected transcripts of all four CO_2_ fixation-related key genes that were already detected in the metagenome analysis. The number of mapped transcripts varied strongly along the environmental gradients (Fig. 1B and 4). Transcripts ranged from a mean transcript copy number/genome of 0 to 4.31 (Fig. 4).

Among all present CO_2_-fixing pathways, the rGlyP key gene showed the lowest transcript abundance along all gradients (Fig. 4B). For some pathways, the high transcript abundance was confined to specific environmental niches. Based on our MAG analysis, we were able to assign function to taxonomy (Fig. 4; Tables S4 to S7).

Transcripts for all key genes were predominantly associated with a limited number of microbial groups ranging from one to five different phyla for each pathway. The rTCA cycle was primarily driven by *Nitrospirota*. Main drivers behind the WLP were the phyla *Desulfobacterota, Nitrospirota*, and *Thermoproteota*. The Calvin cycle was predominantly assigned to the phylum *Pseudomonadota*. Transcripts of the rGlyP at the marine site were mainly assigned to *Chloroflexota* and *Pseudomonadota*. The rGlyP transcripts at the freshwater site were assigned to *Methylomirabilota* and *Desulfobacterota*.

On the transcriptome level, we found three significant correlations (*P* < 0.05) with environmental soil parameters (Fig. 4; Table 3). We observed a significant increase (*P* < 0.05) in the transcript abundance of the WLP key gene with increasing reduction. We found, that with increasing transcript abundance of the rTCA cycle key gene, also the nitrate availability increased significantly (*P* < 0.05). Transcript abundance of the Calvin cycle key gene increased significantly (*P* < 0.05) with higher organic matter availability (Fig. 4; Table 3).

**TABLE 3:** Statistical analysis of the transcript abundance for the key genes of the four present CO_2_-fixing pathways correlating with environmental parameters^*a*^

	Chloride	Organic Matter	Acetate	Pyruvate	Isocitrate	Nitrate	Sulfate	Reduction
Calvin cycle	−0.23	**0.62**	0.22	0.06	0.22	−0.27	−0.13	−0.30
rGlyP	0.04	−0.24	−0.01	−0.07	−0.20	0.24	0.11	−0.24
rTCA cycle	−0.40	0.19	0.22	−0.28	−0.08	**0.67**	−0.30	−0.43
WLP	0.09	0.05	−0.16	0.35	0.05	−0.45	0.05	**0.48**

^*a*^ Significant correlations are indicated by bold numbers (*P* < 0.05). We performed a Spearman’s rank correlation analysis with the transcript abundances of the key genes and eight soil environmental parameters. A positive number indicates increase and a negative number indicates decrease in the gene and transcript abundance with increase in the respective soil parameter. Given numbers represent Spearman's *ρ* (metadata in Table S3).

## DISCUSSION

Phylogenetic and metagenomic analyses identified both heterotrophic and chemo- or photoautotrophic microorganisms. The main orders observed were *Campylobacterales*, *Burkholderiales*, *Flavobacteriales*, and *Anaerolineales*, of which *Campylobacterales*, *Burkholderiales*, and *Anaerolineales* harbor species that are capable of CO_2_ fixation (29–31). Furthermore, it is known that the genetic potential for dark CO_2_ fixation is spread over a broad taxonomic range (33). In general, for all sites, the diversity of 85 different phyla and 185 different classes indicated a highly diverse metabolic potential of the microbiota (Table S2), considering there is a total of 89 bacterial phyla known in the Silva database (34).

To analyze the genetic and transcriptomic potential of dark CO_2_ fixation in Elbe estuary marsh soils, we examined the key genes crucial for the functioning of the respective microbial pathways. Key gene identification is a common concept explored in several previous studies using metagenomics, amplicon sequencing, or qPCR. However, our genome-guided approach questions the validity of using single marker genes to confirm the occurrence, diversity, and abundance of these processes in complex soil environments. Traditionally, the term “marker gene” or “key gene” likely derives from culture-based approaches where light-independent autotrophic growth indicates the necessity of a dark CO_2_ fixation pathway. The identity of a specific dark CO_2_ fixation pathway can be inferred from detecting particular key marker genes. However, the assumption that the presence of these genes indicates light-independent autotrophic growth can be misleading. Although we identified genomes with many marker genes, we could confirm the entire pathway in only a limited number of genomes. The redundancy of these marker genes with other metabolic processes, along with incomplete CO_2_ fixation pathways, necessitates additional scrutiny. This raises concerns about quantitative approaches like qPCR distinguishing between complete and incomplete pathways while still capturing a broad diversity that exists between genomes with complete pathways. Although tools like metaCyc are often used to estimate pathway completeness in incomplete MAGs and have been included in several studies, we did not use this approach in our analysis. These tools can overestimate pathway completeness as 60%–70% of the genes may overlap with other CO_2_ fixation pathways or metabolic processes. Detection thresholds of 75%–80% can lead to scenarios where many pathway-specific genes may be absent while still indicating the genome’s presence. We want to reiterate that these tools are valid for isolates with known physiological data or for initial screenings of many genomes. However, they may be inadequate for complex, often unknown soil metagenomes where key genes overlap across multiple metabolic pathways.

Metagenome and metatranscriptome sequence analyses indicate the functioning of the Calvin cycle, WLP, rGlyP, and rTCA cycle in the marsh soils of the Elbe estuary, suggesting a relevant potential for microbial CO_2_ fixation. Our sampling design covers steep environmental gradients, especially in relation to distance to sea and soil depth. Distance-to-sea gradient was significantly negatively correlated with porewater chloride (i.e., salinity), sulfate, and isocitrate concentrations. Soil depth was significantly positively correlated with the reduction index, reflecting O_2_ deficiency, and negatively with organic matter availability (Table 2; Table S3).

Our hypotheses that (I) increasing salinity and (II) decreasing O_2_ availability increase dark CO_2_ fixation could only partly be confirmed. No pathway shows a significant response to sulfate or chloride concentrations. The strongest albeit not significant response to sulfate and chloride shows the rTCA cycle, which showed nearly no transcript abundance at the marine site (Table 3). In contrast, hypothesis (II) could be confirmed for the WLP that showed a high abundance in samples with a high reduction potential, i.e., low O_2_ availability. The explanation for this lies in the fact that the WLP is a strictly anaerobic pathway (Fig. 4; Table 3 (35, 36)).

Even if the rTCA cycle is restricted to anaerobic or microaerophilic organisms (37), we show that it is not restricted to strictly anaerobic environments that we would expect in the deep soil layers. Our findings indicate a preference of the rTCA cycle to soil surface environments, which are rich in organic matter and have a higher O_2_ availability than the deep soil environments (Fig. 4; Table 3). Another characteristic of the organic-rich high marsh topsoils is the formation of anoxic microsites (38). These areas, characterized by greater organic matter availability, support higher rates of heterotrophic microbial activity and thereby higher CO_2_ concentrations in the soil. In previous studies, the resulting stimulation of CO_2_ fixation was correlated with respiration (33, 39–41). The phylum behind the highest level of transcription of the rTCA was *Nitrospirota*, contributing nearly 100% of the ATP citrate lyase transcripts. *Nitrospira* sp. Palsa-1315 was the dominating genus behind the ATP citrate lyase transcripts (Fig. 4; Table S4). It belongs to the clade B *Nitrospira* (36), and so far, no pure culture of clade B *Nitrospira* exists; therefore, physiological responses to environmental controls need to be further established (42). Our transcriptomic results reveal that the genus *Nitrospira* sp. Palsa-1315 only occurs in brackish to freshwater environments. Further, we observed a significant correlation between the transcript abundance of the reverse tricarboxylic acid (rTCA) cycle key gene (*aclA*) and measured nitrate levels, with nearly all transcripts assigned to the genus *Nitrospira*. This finding is consistent with the known metabolic function of *Nitrospira* species as nitrite oxidizers, which catalyze the conversion of nitrite to nitrate (43). The increased transcript levels of the rTCA cycle in *Nitrospira* suggest a heightened metabolic activity in response to elevated nitrite concentrations, thereby leading to increased nitrate production. This finding suggests that *Nitrospira* simultaneously oxidizes nitrite to nitrate and fixes CO_2_ via the rTCA cycle in the marsh soils of the Elbe estuary. The energy derived from nitrite oxidation facilitates CO_2_ fixation, supporting the autotrophic lifestyle of *Nitrospira*. This dual capability underscores the importance of *Nitrospira* in both the nitrogen and carbon cycles (35, 44).

The gene abundance and transcription for the key enzyme of the Calvin cycle, i.e., RuBisCO, is not strictly dependent on the availability of light although its highest transcript abundance was recorded in two surface layer samples. For other wetland ecosystems, *Burkholderiales* was found to be one of the dominant photosynthetic orders (30). However, in our study, we did not detect any photosynthetic genes (*puf* operon) in the genomes of organisms with the *rbcL* gene. This suggests that the Calvin cycle is not driven by photosynthesis. Rather, we detected sulfur-oxidizing genes in the form of either the thiosulfate dehydrogenase (*tsd*) or the sulfur-oxidizing protein (*soxZ*) in the genomes of nearly all *rbcL*-positive genomes (Fig. 4; Text S1). This indicates that the Calvin cycle is used for chemoautotrophic growth (45). Sulfide oxidation relies on the availability of oxygen but at the same time reducing conditions that enable sulfate reduction to provide sulfide (46). We assume this coupled process of sulfide oxidation and sulfate reduction might be facilitated in organic matter-rich topsoils with anaerobic microsites. Yet, it is surprising that the Calvin cycle transcript abundance exhibited by *Pseudomonadota* is particularly high at the freshwater sites where sulfate availability is lower than in the salt marsh but comparable to the brackish marsh (Table S3). Potentially, greater sulfur cycling despite lower sulfur input in the freshwater marsh is linked to differences in plant community composition among the three marsh sites (47). Marsh rhizospheres are characterized by strong redox oscillations that accelerate coupled sulfate reduction/sulfide oxidation (5, 48).

We observed that transcript abundance for the WLP is linked to the deep soil layers (Fig. 4; Table 3). The data therefore suggest that low O_2_ availability is the driving environmental factor favoring WLP activity because deep soil layers are primarily characterized by low O_2_ concentrations. Recent transcriptome studies in brackish water microbial mats showed that *Desulfobacterota* are capable of expressing the WLP and subsequent acetate and pyruvate metabolism (49). This notion is supported by our taxonomic assignment, which indicates that *Desulfobacterota* play an important role in the transcription of WLP key genes (Fig. 4; Table S5).

### Conclusion and perspective

Our study demonstrates that wetland microbiota are drivers for dark CO_2_ fixation. Wetlands cover only 1% of the Earth’s surface, but store about 20% of the total ecosystem organic carbon stock (50). Their disproportionate role in the global organic carbon budget arises from an imbalance between plant primary production, which adds organic carbon to the soil, and microbial decomposition, which breaks it down (50). In wetlands, microbial decomposition is typically slow due to in general oxygen-poor conditions in the soils. However, the CO_2_ produced through microbial decomposition accumulates, reaching high concentrations under the waterlogged conditions of wetland soils (51, 52). Therefore, dark CO_2_ fixation seems to be of high relevance in wetland soils with potentially important consequences for the climate system. Therefore, our study assessed how the expression of dark CO_2_ fixation in wetland ecosystems varies along environmental gradients within one of the major European estuaries.

Our major findings are (i) only five microbial phyla (*Pseudomonadota*, *Chloroflexota*, *Methylomirabilota*, *Nitrospirota*, and *Desulfobacterota*) are associated with the majority of dark CO_2_ fixation assigned transcripts. (ii) Both community composition and the gene and transcript abundance of CO_2_ fixation-related genes show a high variability in relation to environmental gradients within the estuarine wetland landscape. (iii) Transcript abundance for the WLP seems to be driven by O_2_. (iv) Transcript abundance for the rTCA cycle positively correlates with nitrate, indicating a coupled nitrite oxidation and CO_2_ fixation in *Nitrospira* species. (v) Transcript abundance for the Calvin cycle significantly correlates with organic matter availability. (vi) Variations in the expression of specific functional genes across environmental gradients are more prominent than phylogenetic variations of microbial groups related to dark CO_2_ fixation. These findings suggest that functional diversity plays a crucial role by allowing microorganisms to adapt to their specific environment. This, in turn, leads to niche formation as microorganisms use the specific pathways that make the most efficient use of their ecological potential.

Concerning our metatranscriptomic analyses, it is important to note that although transcriptomes are a good tool to demonstrate the actual transcription of a gene, it must be considered that this is always only a snapshot at the time of sampling. Therefore, in further studies, a temporal component, either short-term to cover the tidal cycle or long-term to cover seasonal effects, should be considered in addition to spatial scales. Our study aimed at understanding the drivers of dark CO_2_ fixation pathways along environmental gradients, and, for the majority of pathways, we succeeded at identifying important environmental controls. However, our study cannot determine the relative importance of dark CO_2_ fixation pathways in terms of counterbalancing ecosystem GHG production. There is an increasing number of studies indicating negative CO_2_ fluxes in the dark for many ecosystem types, such as saline/alkaline soils, dry inland waters, and dry river sediments (53–55). Future molecular research will need to incorporate various biogeochemical tools to understand the relative importance of microbial dark CO_2_ fixation in the climate system.

## MATERIALS AND METHODS

### Soil sample collection

Soil samples were collected from three wetland sites along the salinity gradient of the Elbe estuary (September 2021 [for metagenome and metatranscriptome samples] and September 2022 [for 16S samples]): a salt marsh (N53.927781° E8.914244°), a brackish marsh (N53.834175° E9.371009°), and a freshwater marsh (N53.665624° E9.553203°). Samples were collected with a peat sampler (Eijkelkamp, Giesbeek, Netherlands) to a depth of 50 cm below the soil surface in each of three elevation zones at each site, the high marsh, low marsh, and pioneer zone. To ensure the integrity of the RNA, we immediately placed the samples on dry ice (−78.5°C) upon sampling in the field. Subsequently, soil samples were stored at −80°C until their use for DNA/RNA extraction and metabolite analysis.

### DNA and RNA extraction from soil

DNA was extracted from aliquots of all collected soil samples, which were frozen at −80°C until analysis. Upon thawing on ice, 0.5 g of soil was used for DNA extraction using the NucleoSpin Soil Kit (Macherey-Nagel, Düren, Germany) following the manufacturer’s protocol. Subsequently, the isolated DNA was analyzed at a wavelength of 280 nm using a Nanodrop spectrophotometer (NanoDrop 2000, Thermo Scientific, Waltham, USA). Then, 2 g of soil was used for RNA extraction using the RNeasy PowerSoil Kit (Qiagen, Venlo, Netherlands) following the manufacturer’s protocol. RNA concentrations were quantified using the Qubit 2.0 Fluorometer and the RNA High Sensitivity Assay Kit (RNA HS, Thermo Fisher, Berlin, Germany).

### Determination of organic matter content *via* loss of ignition method

The weight change related to high-temperature oxidation of organic matter was used to determine the organic matter content. About 30–40 g of soil was placed in porcelain crucibles and dried overnight at 60°C in a drying oven. Before ignition, the samples were further dried for 2 h at 105°C in a drying oven to get rid of remaining water. The dry weight (DW) was measured. The soil samples were ignited for 2 h at 500°C in a muffle furnace, cooled in a desiccator, and weighed again. The organic matter content (%) was calculated as follows: OM (%) = (DW after ignition : DW before ignition) * 100

### Metabolite analyses *via* colorimetric assays

Acetate, pyruvate, and isocitrate concentrations were determined by colorimetric assay kits that were used according to the respective manufacturer’s protocol (Sigma; MAK086, MAK071, MAK061). Then, 1 g of soil was mixed with 1 mL of VE H_2_O, followed by ultrasonication and subsequent centrifugation at 11,000×*g* for 1 min. The supernatant was used as sample for metabolite determination.

### Anion analyses *via* ion chromatography

The concentrations of chloride (used as a proxy for salinity), nitrate, and sulfate (considered important alternative electron acceptors) in the soil porewater were approximated using the following method: Frozen soil samples were first thawed, then mixed with deionized water at a ratio of 1 part soil to 10 parts water. The mixture was then filtered through a 0.45 µm syringe filter, and the filtered solution was subsequently analyzed using ion chromatography (883 Basic IC plus, Metrohm, Herisau, Switzerland). Ion concentrations are reported per milliliter of water present in the fresh soil samples after thawing. This calculation is based on the ratio of fresh weight to dry weight of the soil.

### Soil reduction index analyses

Soil reduction, a proxy for oxygen availability, was measured using the indicator of reduction in soils (IRIS) technique (56, 57); here, modified based on Mueller *et al.* and Mittmann-Goetsch *et al.* (39, 58). Briefly, PVC sticks (5 × 70 cm) coated with Fe-oxide paint were inserted into the soil to a depth of 60 cm for 4 weeks. Under reducing conditions, the paint dissolved due to the reduction of Fe(III) to Fe(II). A reduction index (0–1) was then calculated based on the area of paint removal. The sticks were incubated from February 2022 to March 2023, and a yearly average reduction index was used for the specific depth increments of our study. For detailed methods, see Neiske *et al.* (59).

### 16S rRNA gene analyses

Metabarcoding sequencing of 16S rRNA variable regions V3–4 was performed at the Competence Centre for Genomic Analysis, Kiel, Germany using the Illumina Nextera XT Index Kit and primers 341F (5′-CCTACGGGNGGCWGCAG-3′ and 785R (5′-GACTACHVGGGTATCTAATCC-3′) and MiSeq Reagent Kit v3. Adaptor trimming of demultiplexed paired-end reads was performed using Cutadapt (v.4.4) (60). Read filtering and ASV inference were performed using the DADA2 pipeline (v.1.8), with the following specifications (maxEE = 2, maxN = 0, truncQ = 2, truncLen = 260,210). Taxonomic assignment of merged corrected reads was made using the SILVA (v.138.1) database. Downstream analyses were performed using the phyloseq (v.1.41.1) and ggplot2 (3.4.2) R packages.

### Meta-omics sequencing and data processing

Size selection, library preparation, and sequencing of metagenomes were performed at the Ramaciotti Centre for Genomics, Sydney, Australia, using the Ilumina DNA Prep kit and an Illumina NovaSeq 6000 S4 flow cell. Ribosomal RNA depletion, library preparation, and sequencing of metatranscriptomes were performed at the Competence Centre for Genomic Analysis, Kiel, Germany, using the Illumina Stranded Total RNA Prep with Ribo-Zero Plus Microbiome kit and Illumina NovaSeq 6000 S4 Reagent kit.

Our data analysis, which has been previously demonstrated (61, 62), set a new benchmark for metagenomic and metatranscriptomic analyses. Metagenome-assembled genomes (MAGs) represent partial population genomes rather than complete ones, leading to potential inaccuracies in gene and pathway representation. To address this, we constructed a pangenome for each population using multiple MAGs from various samples, allowing us to calculate gene abundances from dereplicated genes while preserving taxonomic information. This approach enables accurate inference of individual gene and pathway abundances without dependence on the completeness of any single MAG. Further, because complex metagenomes can be influenced by the heterogenous occurrence of eukaryotes, viruses, and other genetic elements, rather than normalizing to the size of the library (i.e., FPKM, RPKM metrics), this approach normalizes the abundance of individual genes against prokaryotic single-copy marker genes allowing us to estimate the copies per prokaryotic cell. For specific details on the single-copy marker genes used in this study and their appropriateness see the following references (62–65).

Metagenomic (*n* = 18) and metatranscriptomic (*n* = 18) sequencing data sets were processed as previously described by Paoli *et al.* (61). Briefly, BBMap (v.38.71) was used to quality control sequencing reads from all samples by removing adapters from the reads, removing reads that mapped to quality control sequences (PhiX genome) and discarding low-quality reads (trimq = 14, maq = 20, maxns = 1, and minlength = 45). Quality-controlled reads were merged using bbmerge.sh with a minimum overlap of 16 bases, resulting in merged, unmerged paired, and single reads. The reads from metagenomic samples were assembled into scaffolded contigs (hereafter scaffolds) using the SPAdes assembler (v3.15.2) (66) in metagenomic mode. Scaffolds were length-filtered (≥1,000 bp), and quality-controlled reads from each metagenomic sample were mapped against the scaffolds of each sample. Mapping was performed using BWA (v0.7.17-r1188; -a) (67). Alignments were filtered to be at least 45 bp in length, with an identity of ≥97% and a coverage of ≥80% of the read sequence. The resulting BAM files were processed using the *jgi_summarize_bam_contig_depths* script of MetaBAT2 (v2.12.1) (68) to compute within- and between-sample coverages for each scaffold. The scaffolds were binned by running MetaBAT2 on all samples individually (--minContig 2000 and—maxEdges 500). Metagenomic bins were annotated with Anvio (v7.1.0) (69), quality-controlled using the CheckM (v1.0.13) (70) lineage workflow (completeness ≥50% and contamination <10%) to generate 76,918 MAGs. Complete genes were predicted using Prokka (v1.14.6) (71), and MAGs were taxonomically annotated using GTDB-TK (GTDB-TK v 1.7 and GTDB release R214).

Genes were subsequently clustered at 95% identity, keeping the longest sequence as representative using CD-HIT (v4.8.1) with the parameters -c 0.95 -M 0 -G 0 -aS 0.9 -g 1 -r 0 -d 0 -b 1000. Representative gene sequences were aligned against the KEGG database (release April 2022) using DIAMOND (v2.0.15) (72) and filtered to have a minimum query and subject coverage of 70% and requiring a bitScore of at least 50% of the maximum expected bitScore (reference against itself). Quality-controlled metagenomic and metatranscriptomic sequencing reads were aligned against the set of representative genes. Gene-length normalized read abundances (i.e., gene copies) were first summarized into KEGG orthologous groups (73) and then normalized by the median of single-copy marker genes copies (74) to calculate mean per cell gene and transcript copy numbers of orthologous groups, respectively.

To analyze the abundance of CO_2_ fixing pathways, we combined genomic context and taxonomic information. Our approach to assess the completeness of dark CO_2_ fixation pathways began with identifying genomes containing key genes involved in dark CO_2_ fixation (Tables S4 to S7). We then validated the presence of these pathways by checking for additional critical genes and enzymes. To resolve conflicts between CO_2_ fixation pathways where genes are shared, we compared pathway completeness among closely related genomes at the genus and species levels. Furthermore, a literature review was conducted to address missing genes in known dark CO_2_-fixing taxa and to confirm the identified pathways. Only after ensuring that individual key gene orthologs were representative of organisms capable of dark CO_2_ fixation did we include abundance estimations and subsequent analyses. Detailed criteria for selecting dark CO_2_ fixation key enzymes and considerations at each stage are provided in Supplementary Text 1.

### Statistical analyses

To test the correlations between the environmental parameter chloride, organic matter, acetate, pyruvate, isocitrate, nitrate, sulfate, and reduction index against the key gene and transcript abundance of the CO_2_-fixing pathways, we performed a Spearman’s rank correlation.

## Data Availability

Raw reads of the metagenomic, metatranscriptomic, and 16S rRNA analyses were deposited at the European nucleotide archive ENA under project accession number PRJEB54081 (Biosamples: SAMEA110291994 - SAMEA110292011 and SAMEA113954754 - SAMEA113954807). The gene catalog for the metagenomic and metatranscriptomic analyses and ASV tables for the 16S rRNA analyses were deposited at the ZFDM repository of the University of Hamburg (https://doi.org/10.25592/uhhfdm.13933).

## SUPPLEMENTAL MATERIAL

Supplemental material aem.02177-24-s0001.docx Tables S3 to S7 and Text S1.

Table S1 aem.02177-24-s0002.xlsx Bioinformatic output summary.

Table S2 aem.02177-24-s0003.xlsx List of all phyla and classes (16S).

ASM does not own the copyrights to Supplemental Material that may be linked to, or accessed through, an article. The authors have granted ASM a non-exclusive, world-wide license to publish the Supplemental Material files. Please contact the corresponding author directly for reuse.
