# Supplementary material for: Assessing environmental gradients in relation to dark CO2 fixation in estuarine wetland microbiomes
Source: Appl Environ Microbiol. 2024 Dec 31;91(1):e02177-24. doi: 10.1128/aem.02177-24 (PMC11784365; doi:10.1128/aem.02177-24)
Supplement: Supplemental material — Tables S3 to S7 and Text S1. [file aem.02177-24-s0001.docx]

[added as supplementary Excel file]

**Table S1:** Bioinformatic output summary of metagenome- (METAG) and metatranscriptome (METAT) samples.

[added as supplementary Excel file]

**Table S2:** List of all observed phyla and classes with respective counts in 16S amplicon dataset.

**Table S3:** Environmental parameters along special gradients of the Elbe estuary. The table shows the measured values of the redox index, chloride, nitrate, sulfate, acetate, pyruvate, isocitrate and organic matter content at each of the 18 sampling sites.

**Text S1:**

**Identification of dark CO_2_ fixation in marsh soils**

In the process of aiming to characterize the diversity of dark CO_2_ fixing pathways in sediments of the Elbe estuary we identified a number of limitations to previous approaches. Of these was the acknowledgement that many of the enzymes involved in these pathways are distinguishable from genes involved in heterotrophic processes (namely TCA cycle) or other processes including maintenance of cellular homeostasis. As others have already noted it is therefore necessary to use genes that, if not exclusive, are for the most part strongly associated with a single pathway. Berg et al. (1) previously described a number of “key genes” that can be used as indicators for the presence of individual dark CO_2_ fixing pathways within the genomes of bacteria and archaea. Whilst these genes might be strong indicators of these dark CO_2_ fixing pathways, developing our approach we were made aware that these genes alone are insufficient. We should also acknowledge that our genome-centric metagenome approach, also has limitations in that the recovery of complete metagenome assembled genomes (MAGs) is also limited. More specifically, pathways may appear incomplete due to genes that are otherwise present, but appear to be missing due to differences in assembly and binning.

To this end we sought to develop a pipeline that leverages multiple genes, genomic context, and taxonomic information to assess the completion of dark CO_2_ fixation pathways. Our pipeline generally consisted of:

1. Identifying genomes containing ‘key genes’ involved in dark CO_2_ fixation (Table 1).
2. Validating the occurrence of these pathways by checking for additional genes/enzymes that are critical for the pathway.
3. Resolving conflicts between CO_2_ fixing pathways where one or more genes are shared
4. Validation by comparing pathway completeness for closely related genomes (genus/species)
5. Literature review to resolve missing genes for known dark CO_2_ fixing taxa and to confirm/validate identified pathways.

**Table 1**: List of CO_2_ fixing pathways and affiliated key genes. The selection of key genes based on Berg et al. ([21](#_ENREF_21)) and was supplemented by the malate dehydrogenase for the DC/4-HB cycle and the key genes for the rGly pathway ([22](#_ENREF_22)). Asterisks indicate the key genes selected for analyzing gene and transcript abundance (FIGURE 4) as well as for the correlation analysis of transcript abundance with environmental parameters (TABLE 3).

Only once we were confident that individual ‘key gene’ orthologs were representative of an organism with the capacity to undertake dark CO_2_ fixation did we include the respective abundance estimations and subsequent analyses. Below we will outline in detail our selection criteria for each of the dark CO_2_ fixation pathways and considerations we made at each stage.

Reverse TCA cycle

The rTCA cycle, utilizes many of the enzymes involved in the TCA cycle making its identification difficult using bioinformatic analyses. Fortunately, we observe two enzymes that are markedly different between the forward and reverse cycles, namely the ATP-citrate lyase and the 2-oxoglutarate synthase. In the rTCA cycle the ATP-citrate lyase, replaces the citrate lyase, catalyzing the conversion of citrate to acetyl-CoA and oxalacetate, consuming one molecule of ATP in the process. The ATP-citrate lyase operon comprises two genes, *aclA* and *aclB*, which encode the alpha and beta subunits, respectively. Similarly, the 2-oxoglutarate dehydrogenase, in the rTCA is replaced by the 2-oxoacid:ferredoxin oxidoreductase. Whilst the dehydrogenase utilizes NAD(H), under favorable oxidative conditions, the reverse reaction requires a reduced ferredoxin to overcome the energic constraints. The precise nature of this enzyme appears to differ amongst organisms capable of using the rTCA cycle with some organisms possessing either a 2-subunit or 4-subunit 2-oxoglutarate:ferredoxin oxidoreductase (OGOR, *korAB*(*CD*)), whilst others appear to leverage additional 4-subunit or 5-subunit pyruvate:ferredoxin oxidoreductases (OPOR, *porABCD*(*E*)), with the 5- subunit form proposed to confer addition oxygen tolerance.

We started by identifying genomes containing the ATP-citrate lyase operon, considering genomes containing both the *aclA* and *aclB* genes. We initially identified 15 genomes, associated with six genera, containing either the *aclA* or *aclB* genes, including seven non-redundant *aclA* and seven non-redundant *aclB* orthologs. We identified two *aclA* and one *aclB* othologs amongst four genomes of the *Gammaproteobacteria* genus UBA9214. In one of the four genomes, we actually identified, both *aclA* orthologs, with one clustered alongside other rTCA genes and another embedded in genes associated with DNA replication. We considered this second ortholog to be erroneous and removed it. We identified three genera of Nitrospirota (*Nitrospira*_F, Palsa-1315 and an unclassified NS-4 family member) containg *aclAB* orthologs, with each of the three containing different non-redundant orthologs. The single NS-4 family genome contained both genes, of the three *Nitrospira*_F genomes only one contained both genes, although the individual genes were all homologous. We identified four Palsa-1315 genomes, with two originating from the same sample. These two genomes were assigned to the same genus, both contained both *aclA* and *aclB* but between these two genomes we detected different orthologs of these two genes. A third genome contained a single *aclA* gene which was homologous to one of the two previously mentioned, wheres the fourth genome contained a single *aclB* gene which was not similar to any of the aforementioned. We also detected a single *aclA* ortholog in the genome of a *Desulfobacterota* SM23-61 and a single *aclB* ortholog in the genome of the *Thermoproteota* PALSA-986.

We next sought to validate these occurrences based on the presence of the 2-oxoacid/pyruvate ferredoxin oxidoreductases. We detected the presence of 2-oxoacid ferredoxin oxidoreductases in 432 genomes, mostly in the form of 2-subunit OGOR (*korAB*) but also as 4-subunit OPOR (*porABCD*). Of those genomes with *aclAB* we detected both 2-subunit OGOR and 4-subunit OPOR in the UBA9214, PALSA-986 and SM23-61 genomes. The SM23-61 genome was unique in many ways in that it contained many divergent *korAB* and *porABCD* copies which a pattern that was shared amongst other ATP-citrate lyase lacking *Desulfobacterota* genomes. This uncertainty, the lack of *aclB* was sufficient to exclude this organism from our subsequent analysis. We were unable to detect OGOR (*korAB*) genes amongst any of the *Nitrospirota* genomes, rather amongst these genomes we detected 2-3 clusters encoding three subunit OPOR (*porABC*) with at least one of these clusters containing *porD*. The potential of OPOR enzymes to perform OGOR activity has been previously reported for *Nitrospira* marina and giving us confidence that a similar process is likely occurring here. OPOR genes in *Nitrospira* species were not present on the same contig as the ATP-citrate lyase operon, for a single *Nitrospira*_F genome we found a cluster encoding the ATP-citrate lyase, isocitrate dehydrogenase (*icd*) aconitate hydratase (*acnA*) and succinyl-CoA synthetase beta subunit (*sucC*) providing us confidence that these three *Nitrospira* taxa, similar to *Nitrospira* *marina* are capable of dark CO_2_ fixation via the rTCA cycle. For exploration of gene copy number and transcript abundance we selected the *aclA* gene, representing five orthologs:

**Table S4:** List of *aclA* orthologs and taxonomic assignment.

| *aclA* ortholog (K15230) | Phylum | Class | Family/genus |
| --- | --- | --- | --- |
| STRE22-1_MAGS_0000069813 | Nitrospirota | Nitrospiria | f__NS-4 |
| STRE22-1_MAGS_0000070550 | Nitrospirota | Nitrospiria | g__Nitrospira_F |
| STRE22-1_MAGS_0000070440 | Nitrospirota | Nitrospiria | g__Palsa-1315 |
| STRE22-1_MAGS_0000070441 | Nitrospirota | Nitrospiria | g__Palsa-1315 |
| STRE22-1_MAGS_0000060863 | Pseudomonadota | Gammaproteobacteria | g__UBA9214 |

Wood-Ljungdahl Pathway

The Wood-Ljungdahl pathway involves two specific enzymes namely the Acetyl-CoA synthase (ACS) and a CO dehydrogenase (CODH) forming the CODH/ACS complex. The genes directing the assembly of these two enzyme complexes are often colocalized into a single operon with slight differences between archaea (*cdhA-E*) and bacteria (*acsA-E*). Bacteria encode for an addition THF-corrinoid methyltransferase (*AcsE*) which forms part of the ACS module and archaea encode an additional small subunit (*chdB*) which binds FAD as a cofactor (3).

We commenced our search by identifying genomes with genes encoding the large subunit of the CODH (*cooS/acsA*). This gene alone is not sufficient to identify confirm the presence of the ACS/CODH operon as its homolog *cooS*. Whilst both enzymes are capable of the reduction of CO_2_ to CO or oxidation of CO to CO_2_, the standalone *cooS* enzyme has been shown to be essential for growth on CO suggesting the reverse reaction is favored (4). We identified 47 unique *cooS/acsA* amongst 51 genomes. These genomes primarily represented *Chloroflexota* and *Desulfobacterota* but also included *Myxococcota*, *Nitrospirota*, *Gammaproteobacteria* and *Acidobacteriota*.

We attempted to confirm the presence of the complete *acs/cdh* operons in bacterial and archaeal genomes. The fragmented nature of our MAGs meant that it was not always possible to determine whether missing genes were a consequence of assembly or binning parameters. Incomplete *acs* operons have been demonstrated for instance in *Dehalococcoides*, where rather than CO_2_ fixation the pathway is involved in methyl-tetrahydrofolate recycling supporting methionine biosynthesis (5).

We confirmed the presence of an archaeal type *cdhA-E* operon (across two contigs) in the genome of *Bathyarchaeia* (g__PALSA-986), a complete bacterial type (*acsA-E*, 3 contigs) operon in the genome of a *Nitrospirota* (g__JAAXXJ01), partial bacterial type (*acsACD*) in genome of *Nitrospirota* g__JACRPZ01, partial bacterial type (*ascBCDE*) in genome of Desulfobacterota (g__BM002), partial bacterial type (*ascCDE*) in genome of *Desulfobacterota* (g__BM002), partial bacterial type (*ascADE*) in genome of *Desulfobacterota* (g__SM23-61), partial bacterial type (*ascABCE*) in genome of *Desulfobacterota* (SM23-61), partial bacterial type (*ascBCE*) in 2 genomes of *Desulfobacterota* (g__JAHJIQ01), partial bacterial type (*ascABCE*) in five genomes of *Desulfobacterota* ( g__BM004), partial bacterial type (*ascABE*) in genome of *Desulfobacterota* ( g__ g__UBA2230), partial bacterial type (*ascABCE*) in genome of *Desulfobacterota* (g__UBA11574 ), partial bacterial type (*ascABCE*) in genome of *Desulfobacterota* (g__ g__JAAXQD01), partial bacterial type (*ascABCE*) in 5 genomes of *Desulfobacterota* (g__DSXZ01), partial bacterial type (*ascABCE*) in genome of *Desulfobacterota* (g__4484-190-2), partial bacterial type (*ascACDE*) in genome of *Desulfobacterota* (g__ g__ partial bacterial type (*ascABCE*) in genome of *Desulfobacterota* (g__S015-6)), 3 genomes only *acsAC* g__SpSt-501.

Amongst the *Desulfobacterota* we observed a consistent pattern amongst the different taxa, specifically members of the *Syntrophobacteria* lacked *acsA*, SM23-61 lacked *acsB-C* and whereas other genomes consistently lacked *acsD*. The lack of *acsA* in amongst *Syntrophobacteria* is consistent with that of other potentially syntrophic bacteria e.g. *Dehalococcoides* which runs the WLP in reverse cleaving acetyl-CoA and leading to the accumulation of CO as an inhibitory by-product. For those lacking *acsD*, this included the *Desulfobulbia* which represent the enigmatic cable-bacteria. There is a general consensus for the presence of complete and functional WLP, with studies highlighting the capacity of *Desulfobulbia* to grow autotrophically in the absence of an organic carbon source. This discrepancy might arise due to differences in annotation approaches, with one study in particular noting that the WLP was incomplete when annotated with KEGG but then complete using UniProtKB annotation. Manual inspection of contigs containing all genes, except *acsD*, showed no evidence for an unannotated gene. Despite this, considering the overwhelming literature evidence in support of autotrophic growth amongst *Desulfobulbia* we concluded this consistent lack of an annotated *acsD* was insufficient to discount that there is a high likelihood that the WLP is active in these genomes.

For exploration of gene copy number and transcript abundance we considered which of the four genes shared between archaea and bacteria would be suitable. We immediately discounted *acsD*/*cdhD* as it was absent from the genomes of *Desulfobulbia*. We also ruled out *acsA*/*cdhA* as its homology with *cooS* meant it was often duplicated within the genome. Ultimately, we chose *acsC*/*cdhE* as it was nearly always exclusively present at single copy in those organisms with a near complete operon and was well conserved amongst closely related genomes.

This resulted in 13 *acsC*/*cdhE* orthologs that we used for subsequent analyses:

**Table S5:** List of *acsC/cdhE* orthologs and taxonomic assignment.

| *acsC/cdhE* ortholog (K00197) | Phylum | Class | Family/genus |
| --- | --- | --- | --- |
| STRE22-1_MAGS_0000172077 | Desulfobacterota | DSM-4660 | g__4484-190-2 |
| STRE22-1_MAGS_0000171191 | Desulfobacterota | DSM-4660 | g__DSXZ01 |
| STRE22-1_MAGS_0000173103 | Desulfobacterota | Desulfobacteria | g__JAAXQD01 |
| STRE22-1_MAGS_0000173477 | Desulfobacterota | Desulfobacteria | g__SpSt-501 |
| STRE22-1_MAGS_0000173498 | Desulfobacterota | Desulfobacteria | g__SpSt-501 |
| STRE22-1_MAGS_0000172741 | Desulfobacterota | Desulfobacteria | g__UBA11574 |
| STRE22-1_MAGS_0000167657 | Desulfobacterota | Desulfobulbia | g__BM004 |
| STRE22-1_MAGS_0000167616 | Desulfobacterota | Desulfobulbia | g__BM004 |
| STRE22-1_MAGS_0000167621 | Desulfobacterota | Desulfobulbia | g__BM004 |
| STRE22-1_MAGS_0000172603 | Desulfobacterota | Desulfobulbia | g__JABDQA01 |
| STRE22-1_MAGS_0000154618 | Desulfobacterota | Desulfobulbia | g__JAHJIQ01 |
| STRE22-1_MAGS_0000173451 | Nitrospirota | UBA9217 | g__JAAXXJ01 |
| STRE22-1_MAGS_0000144585 | Thermoproteota | Bathyarchaeia | g__PALSA-986 |

Calvin Cycle

The Calvin cycle represents the mechanism for inorganic carbon fixation by all photosynthetic organisms. RuBisCO is the central enzyme in this catalyzing the carboxylation of ribose-1,5-bisphophsate with CO_2_. We identified 99 MAGs containing the gene encoding the RuBisCO large subunit (*rbcL*) and 86 MAGs which contained, in addition, the gene encoding the RuBisCO small subunit (*rbcS*). Considering only Type I RuBisCO possesses the small subunit, compared to type II-III which also perform carbon fixation (6), we did not consider absence of *rbcS* as disqualifying. Therefore, we also considered additional genes, specifically we insisted that the phosphoribulokinase (*prkB*) was present in addition to both *rbc* genes. In instances where *prkB* or one of the *rbc* genes was absent we additional checked for other genes in the Calvin cycle specifically phosphoglycerate kinase (*pgk*) and ribose 5-phosphate isomerase (*rpiAB*).

We did not detect any photosynthetic genes (*puf* operon) in the genomes of those organisms with rbcL suggesting that the Calvin cycle is not driven by photosynthesis. Rather we detected sulfur oxidizing genes in the form of either thiosulfate dehydrogenase (*tsd*) or sulfur oxidizing protein *SoxZ* in the genomes of nearly all *rbcL* positive genomes. We also identified bacterioferritin gene (*bfd*/*bfr*) in a number of *rbcL* positive genomes, but also those lacking *rbcL*. What was more interesting is that for a number of genomes (e.g. UBA9214) bacterioferritin was colocalized with *rbcL*/*S* on the genome. In contrast, we did not detect a strong association of Fe(II) oxidizing or ammonium oxidizing pathways in the *rbcL* positive genomes.

In contrast to other pathways, we identified multiple distinct copies of the *rbcL*, *rbcS* and *prkB* genes. Ultimately, we decided to retain *rbcL* abundances for downstream analyses. In a few cases we detected more than distinct copy of the *rbcL* gene per genome, with one clearly colocalized with *rbcS* and other carbon fixing genes. In these few instances we retained only the colocalized *rbcL* copy.

This resulted in 38 *rbcL* orthologs that we used for subsequent analyses:

**Table S6:** List of *rbcL* orthologs and taxonomic assignment.

| *rbcL* ortholog (K01601) | Phylum | Class | Family/genus |
| --- | --- | --- | --- |
| STRE22-1_MAGS_0000139357 | Acidobacteriota | Vicinamibacteria |  |
| STRE22-1_MAGS_0000187619 | Chloroflexota | Anaerolineae |  |
| STRE22-1_MAGS_0000129077 | Chloroflexota | Limnocylindria | g__CF-167 |
| STRE22-1_MAGS_0000132891 | Methylomirabilota | Methylomirabilia | g__Methylomirabilis |
| STRE22-1_MAGS_0000133626 | Methylomirabilota | Methylomirabilia | g__Methylomirabilis |
| STRE22-1_MAGS_0000133639 | Methylomirabilota | Methylomirabilia | g__Methylomirabilis |
| STRE22-1_MAGS_0000134692 | Myxococcota_A | UBA9160 |  |
| STRE22-1_MAGS_0000146838 | Pseudomonadota | Alphaproteobacteria | g__JADFVY01 |
| STRE22-1_MAGS_0000133783 | Pseudomonadota | Alphaproteobacteria | g__R-RK-3 |
| STRE22-1_MAGS_0000128945 | Pseudomonadota | Gammaproteobacteria |  |
| STRE22-1_MAGS_0000129166 | Pseudomonadota | Gammaproteobacteria |  |
| STRE22-1_MAGS_0000158893 | Pseudomonadota | Gammaproteobacteria |  |
| STRE22-1_MAGS_0000131940 | Pseudomonadota | Gammaproteobacteria | g__CADEEN01 |
| STRE22-1_MAGS_0000132721 | Pseudomonadota | Gammaproteobacteria | g__Ga0077527 |
| STRE22-1_MAGS_0000122682 | Pseudomonadota | Gammaproteobacteria | g__Gallionella |
| STRE22-1_MAGS_0000130040 | Pseudomonadota | Gammaproteobacteria | g__GCA-001735895 |
| STRE22-1_MAGS_0000145946 | Pseudomonadota | Gammaproteobacteria | g__GCA-001735895 |
| STRE22-1_MAGS_0000148759 | Pseudomonadota | Gammaproteobacteria | g__GCA-001735895 |
| STRE22-1_MAGS_0000141422 | Pseudomonadota | Gammaproteobacteria | g__JAABQT01 |
| STRE22-1_MAGS_0000151976 | Pseudomonadota | Gammaproteobacteria | g__JAACFB01 |
| STRE22-1_MAGS_0000158726 | Pseudomonadota | Gammaproteobacteria | g__JAACFE01 |
| STRE22-1_MAGS_0000158436 | Pseudomonadota | Gammaproteobacteria | g__JABDPF01 |
| STRE22-1_MAGS_0000128793 | Pseudomonadota | Gammaproteobacteria | g__RPQJ01 |
| STRE22-1_MAGS_0000128062 | Pseudomonadota | Gammaproteobacteria | g__SG8-30 |
| STRE22-1_MAGS_0000142226 | Pseudomonadota | Gammaproteobacteria | g__SG8-39 |
| STRE22-1_MAGS_0000129882 | Pseudomonadota | Gammaproteobacteria | g__SM1-46 |
| STRE22-1_MAGS_0000127155 | Pseudomonadota | Gammaproteobacteria | g__Sulfuricaulis |
| STRE22-1_MAGS_0000129631 | Pseudomonadota | Gammaproteobacteria | g__Sulfuricaulis |
| STRE22-1_MAGS_0000143834 | Pseudomonadota | Gammaproteobacteria | g__Sulfuricaulis |
| STRE22-1_MAGS_0000157466 | Pseudomonadota | Gammaproteobacteria | g__SZUA-36 |
| STRE22-1_MAGS_0000158405 | Pseudomonadota | Gammaproteobacteria | g__SZUA-36 |
| STRE22-1_MAGS_0000146986 | Pseudomonadota | Gammaproteobacteria | g__Thiogranum |
| STRE22-1_MAGS_0000155953 | Pseudomonadota | Gammaproteobacteria | g__UBA1847 |
| STRE22-1_MAGS_0000127856 | Pseudomonadota | Gammaproteobacteria | g__UBA6901 |
| STRE22-1_MAGS_0000131669 | Pseudomonadota | Gammaproteobacteria | g__UBA9214 |
| STRE22-1_MAGS_0000145819 | Pseudomonadota | Gammaproteobacteria | g__UBA9214 |
| STRE22-1_MAGS_0000146716 | Pseudomonadota | Gammaproteobacteria | g__UBA9214 |
| STRE22-1_MAGS_0000146998 | Pseudomonadota | Gammaproteobacteria | g__UBA9214 |

Reductive Glycine Pathway

The reductive glycine pathway (rGlyP) commences in a similar manner to the Wood-Lungdahl pathway with the reduction of CO_2_ to formate, however following the reduction of formate rather than CO, condensation of a second CO_2_ occurs giving rise to glycine. The critical enzymes in this step are the methyenyl-THF cyclohydrolase (MTHFD) involved in the reduction of formate, glycine cleavage/synthase (*glr*) which performs the condensation reaction of the second CO_2_ molecule to give rise to glycine and the glycine reductase (*grd*) which converts glycine to acetyl-P.

The glycine reductase complex is only partially specific to the reductive glycine pathway, in that homologs are also known to act to reduce also sarcosine and betaine. The enzyme complex comprises four subunits with *grdA*, *grdC* and *grdD* involved in none specific reactions, however *grdB*/*E* and its paralogs *grdF*/*G* and *grdH*/*I* confer specificity of the different substrates. As for other pathways we sought to establish the presence of the *grd* operon in genomes also containing the MTHFD and glycine cleavage/synthase module. In total we identified 14 *grdB* orthologs. Of these four did not occur alongside other *grdB* genes, the MTHFD or *glr* genes. Eight of the remaining ten orthologous *grdB* copies represented four pairs of co-occurring and often adjacent genes, consisting of a > 1000 bp gene and a ~230-250 bp gene. The combined size of these two fragments were consistent with database and literature searches for this gene. These genes were situated between the *grdA* and a gene annotated as glycine C-acetyltransferase consistent with the function of *grdC*. It was not clear what contributed to this cleavage, however we opted to select in each case the larger fragment for quantification.

**Table S7:** List of *rbcL* orthologs and taxonomic assignment.

| *grdB* ortholog (K10672) | Phylum | Class | Family/genus |
| --- | --- | --- | --- |
| STRE22-1_MAGS_0000296067 | Pseudomonadota | Alphaproteobacteria | g__ |
| STRE22-1_MAGS_0000303920 | Chloroflexota | Anaerolineae | g__J095 |
| STRE22-1_MAGS_0000306728 | Methylomirabilota | Methylomirabilia | g__ |
| STRE22-1_MAGS_0000311329 | Desulfobacterota_B | Binatia | g__JACPFE01 |

Hydroxybutarate cycles (DC/4-HB cycle, 3-HP/4-HB cycle, 3-HP-bicycle)

The decarboxylative 4-hydroxybutarate and 3-hydroxypropinoate/4-hydroxyburate cycles share a number of common enzymes, specifically involved in the regeneration of acetyl-Coa from succinyl-CoA via 4-HB-CoA, crotonoyl-CoA intermediates. The pathways specifically diverge with the DC/HB-cycle mirroring the rTCA cycle with the carboxylation of acetyl-CoA to pyruvate and reduction via malate and fumarate intermediates. In contrast the 3-HP/4-HB cycle mirrors the 3-HP bicycle with carboxylation of acetyl-CoA to a malonyl-CoA intermediate which is reduced via 3-HP/3-HP-CoA and methylmalonyl-CoA intermediates.

Considering the number of shared genes/enzymes between these pathways, their overlap with other carbon-fixing pathways and the potential redundancy of genes with other metabolic pathways, ultimately proving the functionality of these pathways from metagenome data is tenuous. Despite this we still sought to identify genomes, where these pathways are present and use this data to explore their ecological significance.

We commenced our search by identifying 94 genomes containing the gene encoding the 4-hydroxybutyryl-CoA dehydratase (*abfD*). These 94 genomes included *Chloroflexota*, *Desulfobacterota*, *Myxococcota* as well as *Gamma-* and *Alphaproteobacteria* and comprised 60 unique *abfD* orthologs. We next expanded our search to include additional genes involved in the 3-HP/4-HB cycle namely the methylmalonyl-CoA mutase (MUT) which is also present in the 3-HP bicycle and the 3-methylcrotonyl-CoA carboxylase which is also shared by the DC/4-HB cycle. We only identified three genomes, belonging to the *Alphaproteobacterial* genus WHUA01 which contained all three of these genes. We did not detect either the phosphoenolpyruvate (PEP) carboxylase (*ppc*), which would be necessary for a functional DC/HB cycle. However, when we expanded our search to also include genes involved in 3-HP metabolism we were surprised to find a complete lack of critical genes. Most notably enzymes (ec:6.4.1.2, ec:1.2.1.75, ec:1.1.1.298, ec:6.2.1.36) involved in carboxylation of acetyl-CoA to propionyl-CoA via 3-HP were completely absent from the dataset excluding (ec:4.2.1.116, and ec:1.3.1.84), which occurred in a handful of genomes with no overlap.

Based on the complete absence of these enzymes from any genome we considered that both the 3-HP/4-HB cycle and the 3-HP bicycle were completely absent from genomes in our samples.

We next refined our list of *abfD* containing genomes to those that do contain the PEP carboxylase as a means for identifying those organisms utilizing the DC/4-HB cycle. We identified seven genomes belonging to the *Gammaprotoebacterial* genus g__CADEEN01, one genome from an unclassified *Desulfuromonadia* (STRE22-1_SAMEA110292004_MAG_00000068), one genome from the *Desulfobacterota* g__S015-6, one genome from an *Acidimicrobiia* genus UBA4744 that contained both *abfD* and *ppc* genes. However, despite containing the *abfD* and *ppc* genes, we were unable to detect the presence of critical enzymes in any of these genomes, that would allow for the reduction of succinyl-CoA to acetyl-CoA via 4-HB. Specifically, were did not identify any succinyl-CoA reductases, succinate semialdehyde reductases, or enoyl-reductases. This strongly suggests that despite the diversity of *abfD* genes in our dataset very few are involved directly in dark CO_2_ fixing pathways.

A recent analysis examining the occurrence of dark CO_2_ fixing pathways across several thousand MAGs suggested that the DC/4-HB cycle appears to be restricted to archaea, specifically *Sulfolobales* and *Thermoproteales*, whereas the 3-HP/4-HB cycle occurs in both archaea as well as *Alpha-* and G*ammaproteobacteria*. 3-HP bicycle genes were first described for *Chloroflexota* but were also found in the aforementioned study also in *Gammaproteobacteria* and *Gemmatimonadota*. 3-HP CO_2_ fixation pathways (3-HP/4-HB and 3-HP bicycle) were generally limited to photosynthetic bacteria with pathways for bacteriochlorophyll biosynthesis, an aspect which was also lacking from any of our sequenced genomes (7).
